# Diverse phylogenetic neighborhoods enhance community resistance to drought in experimental assemblages

**DOI:** 10.1038/s41598-021-01991-z

**Published:** 2021-11-18

**Authors:** Rocío Chaves, Pablo Ferrandis, Adrián Escudero, Arantzazu L. Luzuriaga

**Affiliations:** 1 Department of Biology and Geology, Rey Juan Carlos University, C/Tulipán s/n, 28933 Móstoles, Madrid Spain; 2 Botanic Institute of the University of Castilla-La Mancha, Castilla-La Mancha Botanic Garden, Avda. de La Mancha s/n, 02006 Albacete, Spain

**Keywords:** Plant ecology, Community ecology

## Abstract

Although the role played by phylogeny in the assembly of plant communities remains as a priority to complete the theory of species coexistence, experimental evidence is lacking. It is still unclear to what extent phylogenetic diversity is a driver or a consequence of species assembly processes. We experimentally explored how phylogenetic diversity can drive the community level responses to drought conditions in annual plant communities. We manipulated the initial phylogenetic diversity of the assemblages and the water availability in a common garden experiment with two irrigation treatments: average natural rainfall and drought, formed with annual plant species of gypsum ecosystems of Central Spain. We recorded plant survival and the numbers of flowering and fruiting plants per species in each assemblage. GLMMs were performed for the proportion of surviving, flowering, fruiting plants per species and for total proportion of surviving species and plants per pot. In water limited conditions, high phylogenetic diversity favored species coexistence over time with higher plant survival and more flowering and fruiting plants per species and more species and plants surviving per pot. Our results agree with the existence of niche complementarity and the convergence of water economy strategies as major mechanisms for promoting species coexistence in plant assemblages in semiarid Mediterranean habitats. Our findings point to high phylogenetic diversity among neighboring plants as a plausible feature underpinning the coexistence of species, because the success of each species in terms of surviving and producing offspring in drought conditions was greater when the initial phylogenetic diversity was higher. Our study is a step forward to understand how phylogenetic relatedness is connected to the mechanisms determining the maintenance of biodiversity.

## Introduction

The current theoretical framework and evidence suggest that both stochastic^1,2^ and deterministic mechanisms^3–7^ operate simultaneously on the assembly of plant communities^8–11^. Abiotic and biotic filters—mostly acting at the regional and the fine spatial scales, respectively—are important drivers of species assembly in drylands^12^, together with facilitation that has been described as an important coexistence mechanism in stressful environments (i.e., the Stress Gradient Hypothesis, sensu Bertness and Callaway^13^). Plant trait-based community ecology is recognized as an invaluable tool to understand these processes because it provides morphological or physiological trait-based indices in order to identify the role played by each species at the community level in a niche complementarity context^14^. Thus, a species will become part of a realized species assemblage only if it possesses suitable traits to pass through the filters imposed by restrictive environmental conditions and it reduces niche overlap with neighbor species^15^. In the last two decades, the toolbox of community ecologists has incorporated analyses of the phylogenetic patterns of plant communities to understand assembly processes^16,17^. It is evident that historical and evolutionary mechanisms related to migration and speciation are critical for the formation of the regional species pool, but it is not clear how the phylogenetic diversity that describes the degree of relatedness among species can provide information about assembly processes that occur at the ecological time scale^5,18^. A phylogeny should summarize the ecological requirements of coexisting species because it synthesizes the morphological, physiological, and phenological changes in each species throughout evolutionary time in a reduced geographical domain^19–21^. However, phylogenetic distance among species could indicate not only niche differences, but also competitive inequalities (differences in species competitive abilities) which should drive competitive exclusion^22,23^. Indeed, the identification of niche differences should be even more feasible throughout the phylogenetic than the functional approach^14,24^, because the latter would require the analysis of several traits most of which might be hard or impossible to measure^16,25^. Thus, phylogenetic diversity could represent more reliably niche differences than functional diversity^26–30^, but see Ref.^31^).

Many studies have aimed to detect assembly mechanisms based on the observed phylogenetic diversities under field conditions (i.e., phylogenetic response)^16,23,32–34^, but the results are not completely coherent and they do not indicate unambiguous relationships among phylogenetic diversity and assembly processes^18,19^. For instance, coexistence of phylogenetically close species is usually interpreted as a result of habitat filtering processes and can be indicative of habitat use as a conserved trait along phylogeny^16,35^. However, these types of low phylogenetic diversity assemblages can also result from competition among species when the competitive ability under certain environmental conditions is associated with whole clades^33^. By contrast, high phylogenetic diversity responses could be associated with facilitation among species^35,36^, but also with competition processes when competitive exclusion occurs between close relatives with patent niche overlap^16,37,38^. Furthermore, if niche convergence occurs among distantly related taxa, high phylogenetic diversity will also be observed in the resulting species assemblages under competitive scenarios^39^.

Consequently, progress needs to be made in order to elucidate the causal relationships among phylogenetic diversity and assembly mechanisms by directly manipulating the phylogenetic diversity of whole assemblages (i.e., phylogenetic effect) together with the abiotic and biotic conditions. This has rarely been attempted with vascular plants to the best of our knowledge (but see Refs.^40,41^). A wide consensus exists on the need for experimental approaches to specifically analyze the mechanisms involved in the assembly of plant communities^5,6^. Ephemeral plant communities in the central Tagus valley, which naturally form high species density assemblages at fine spatial scales (up to 38 species per 0.25 m^2^ in rainy years; see Refs.^12,44^) and a rich regional species pool comprising around 120 annual plant species are especially appropriate for these type of experiments^42,43^ In addition, the small size of individuals (average height = 10 cm) and their short and synchronized life cycles (from autumn to spring) makes it feasible to manipulate the entire community and establish species assemblages of known phylogenetic structure. These features allow the design and implementation of experimental communities containing selected species under controlled conditions in common gardens^42^.

Shifts of assembly mechanisms in a regional species pool greatly depend on the harshness of the abiotic conditions^45^, especially dealing with resource availability^12,46^. Since water availability is the main limiting resource in semi-arid Mediterranean ecosystems^47^, it strongly affects plant community dynamics^48^, particularly species richness and composition^12^. Furthermore, species-specific interactions (i.e., competition and facilitation) that strongly determine the assembly of species assemblages (e.g., Ref.^49^) can shift depending on water availability^12,50^. In the present study, we manipulated both the level of phylogenetic relatedness among coexisting plants (i.e., phylogenetic effect) and the level of irrigation in a common garden experiment by reproducing realistic annual plant assemblages along a period that encompasses a complete life cycle of annual plants (see “Methods”). We aimed to evaluate the effects of the phylogenetic diversity of assemblages on surrogates of community performance (i.e., surviving species, flowering plants, fruiting plants) under different water availability scenarios (i.e., stressful levels). In the coexistence theory context^6^, community performance is the net sum of all the differences in fitness of the species that form an assemblage^51^. The fitness inequalities among species may cause some of them to disappear, and thus the decrease in the number of species per sampling unit registered throughout the experiment indicated the limitations imposed by the experimental treatments.

**Figure 1:**
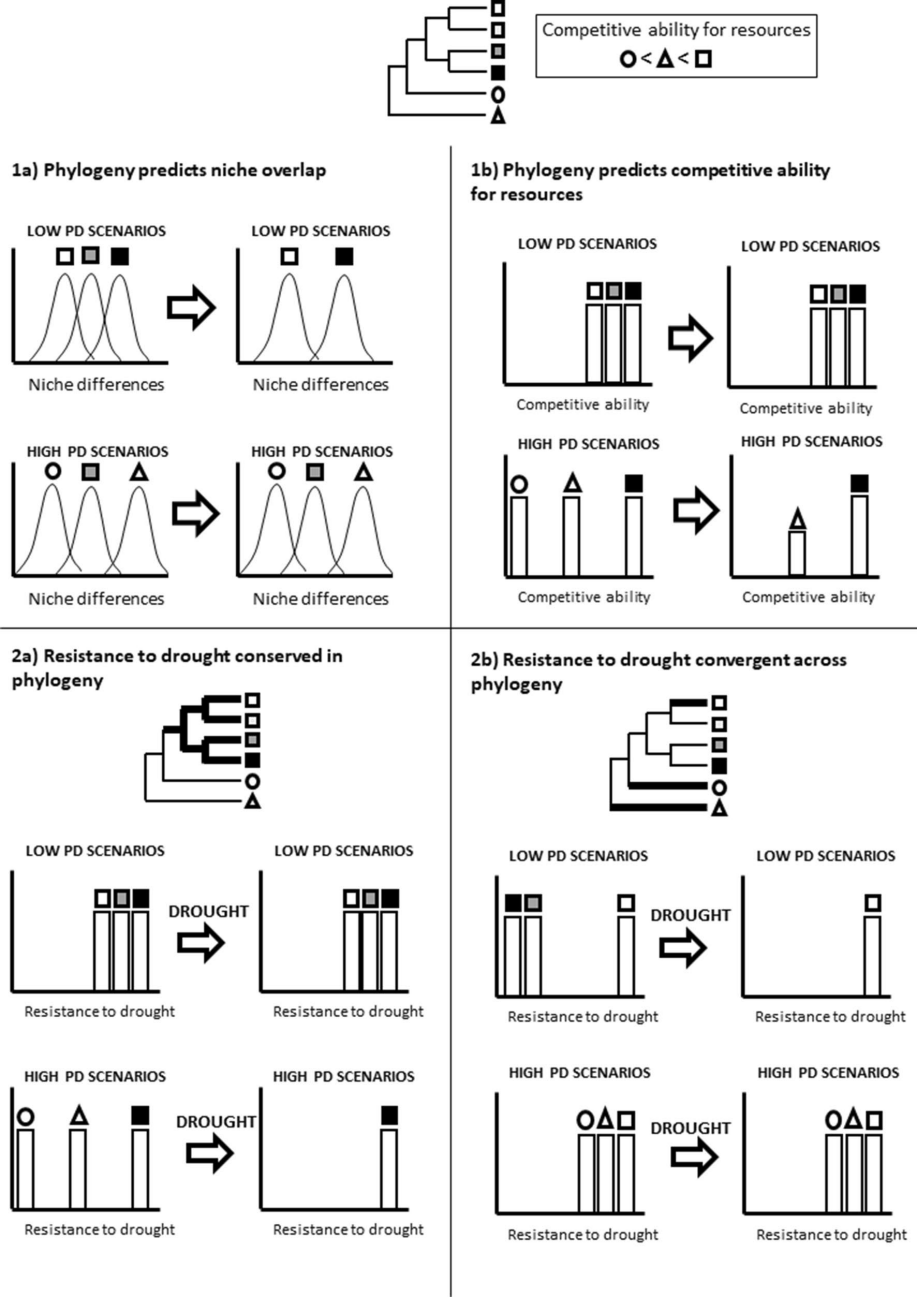
Conceptual model illustrating the hypotheses on the mechanisms involved in the assembly of the annual plant community related to phylogenetic diversity. (1) If phylogenetic relatedness predicts the intensity of niche overlap-differentiation among species, then plants will coexist more readily in high phylogenetic diversity scenarios due to functional/niche complementarity. Conversely, if phylogenetic relatedness predicts the competitive ability of species, then coexistence will be more likely to occur in low phylogenetic diversity scenarios (i.e., competition symmetry will enhance the coexistence among similar competitors). (2) If functional traits related to water economy are phylogenetically conserved, the response of plants to drought would be more heterogeneous in high diversity assemblages, resulting in a faster decline of species richness. In contrast, if water economy traits in the species pool are convergent among distantly related taxa, phylogenetically diverse assemblages will be more resistant to drought than those formed by close relatives.

The two main hypotheses tested in this study are (see our conceptual framework in Fig. 1): (1) If phylogenetic relatedness at the beginning of the growing season predicts niche differences among species, then plants will coexist more readily in high phylogenetic diversity scenarios due to functional/niche complementarity. By contrast, if phylogenetic relatedness predicts the competitive ability of species, in the manner that closely related species can compete more efficiently for the same resources^16^, then species will be more likely to coexist in low phylogenetic diversity scenarios. Previous studies have suggested that the competition among closely related species is symmetric, i.e., competition intensity between close relatives is very similar for both competitors^52,53^, and thus it does not cause exclusion, which can enhance the coexistence among similar competitor species^54^. (2) Provided that the functional traits related to water economy are phylogenetically conserved, the effect of drought on the community and species level performance will be less intense in assemblages containing more resistant clades. Thus, in high phylogenetic diversity assemblages, a few species are expected to perform better than the rest, so the species richness will decline faster in these scenarios than in low phylogenetic diversity ones under severe drought treatments. By contrast, if the functional traits related to water economy are convergent among distantly related taxa, then we expect phylogenetically diverse assemblages to be more resistant to drought than those that are closely related. Finally, if drought resistance would randomly occur along phylogeny, we expect that the response of species assemblages to water limitation would not show a clear pattern in different experimental scenarios.

## Materials and methods

The target plant community comprised annual plant communities on gypsum soils in the Tagus valley, central Spain, which has a semiarid Mediterranean climate with mean annual temperatures around 14.5 °C and mean annual precipitation of 400 mm m^–2^ year^–1^. Precipitation events occur mainly in the late autumn and early spring, and there is an intense summer drought (Aranjuez weather station, 40° 4′ 2″ N; 3° 32′ 46″ W, 540 m). The dominant vegetation comprises gypsophilous dwarf shrubs (e.g., *Lepidium subulatum* L., *Centaurea hyssopifolia* Vahl, *Gypsophila struthium* L., *Helianthemum squamatum* (L.) Dum. Cours., *Thymus lacaitae* Pau, *Herniaria fruticosa* L., and *Frankenia thymifolia* Desf.) scattered in a matrix of bare soil covered mostly with a biological soil crust and seasonal cover of annual plants. The annual plant communities are formed from a rich regional floristic pool (over 120 species in the middle Tagus valley^43^) of ephemeral, highly life-cycle synchronized plants (October–early June), generating high species density assemblages at fine spatial scales (up to 38 species per 0.25 m^2^ in favorable years^12,44^).

From March to June during 2016 and 2017, we collected seeds from more than 40 individuals of 60 annual plant species that naturally co-occur in open areas in the field in three nearby locations (Aranjuez (40° 02′ 11.7″ N, 3° 32′ 59.5″ W; 591 m), Ciempozuelos (40° 08′ 36.9″ N, 3° 37′ 00.0″ W; 585 m), and Portalrubio de Guadamejud (40° 17′ 34.4″ N, 2° 35′ 31.0″ W; 755 m). Seeds were cleaned and submitted to a light hot thermal shock (15 days at 50 °C) to simulate hot summer conditions to break the seed dormancy. We established 6 experimental scenarios, but we finally maintained 4 of them because two of the scenarios did not fulfill the requirements to enter the experiment (seed germination was not enough at each plot), thus, we finally used 28 species to build the species assemblages (see below). We prepared a common garden experiment with 110 experimental assemblages and more than 7000 seedlings.

**Figure 2:**
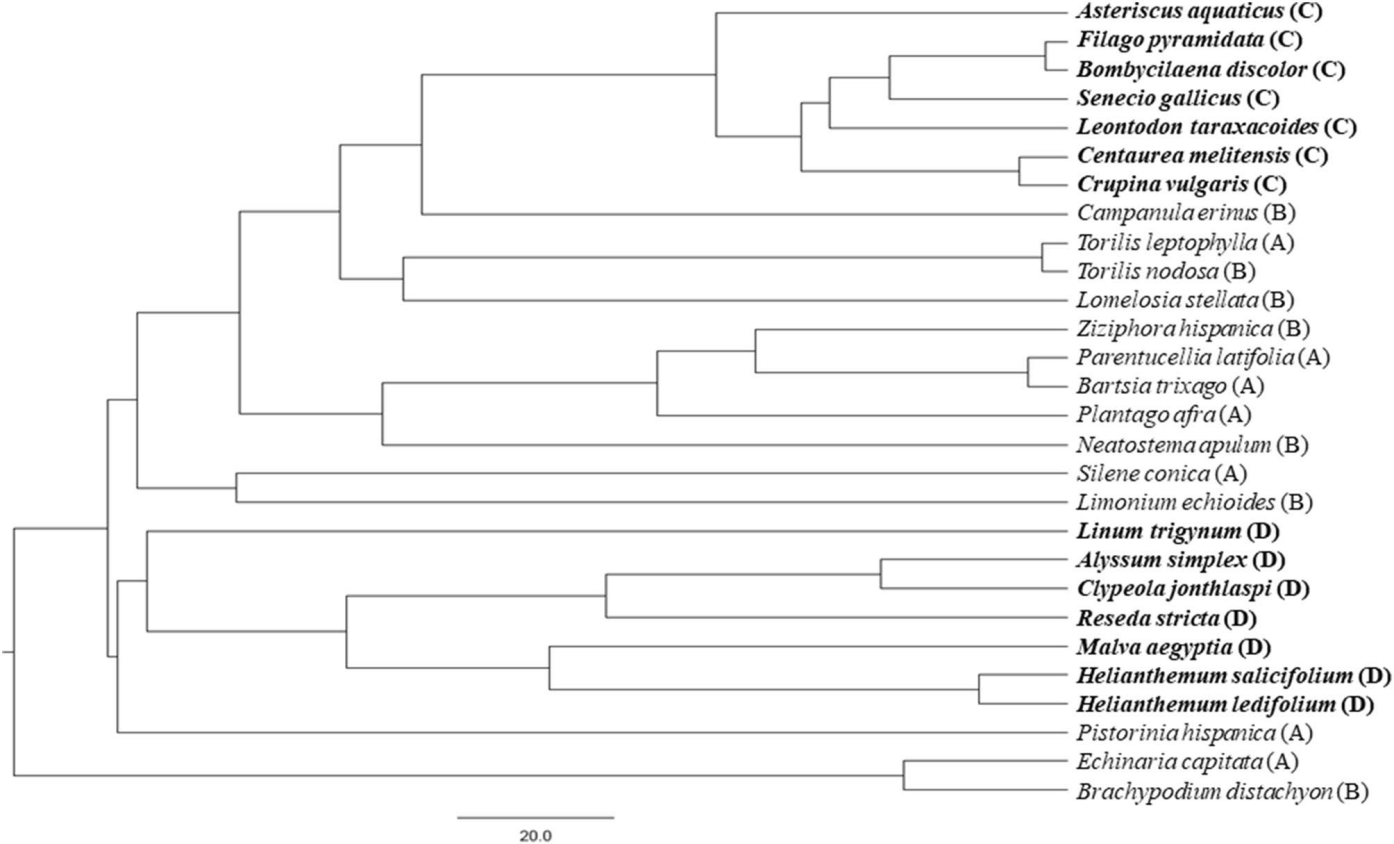
Distance-based phylogenetic tree for the 28 annual plant species used to prepare the experimental scenarios. Based on “V.PhyloMaker” package in R. The capital letters between brackets next to the names of species indicate the species combinations in which they participated. In nonbold typeface, the high phylogenetic diversity scenarios (A and B combinations); in bold, the low phylogenetic diversity scenarios (C and D combinations).

The experimental design consisted of manipulating the phylogenetic diversity of starting experimental assemblages together with water availability treatments. The plant emergence of species in these communities is highly synchronized, so we prepared different phylogenetic combinations at this early demographic stage for our experimental treatments. In order to select the high and low phylogenetic diversity scenarios, we calculated the phylogenetic species variability (PSV) index because it is bounded between 0 and 1 and it is easily interpretable (PSV ~ 1: high phylogenetic diversity; PSV ~ 0: low phylogenetic diversity)^55^ and the SES.MPD index^56^. The SES.MPD is a standardized phylogenetic index that contrasts the observed Mean Pairwise Distance (MPD) to 1000 null assemblages calculated over subsets of random species in the local phylogenetic tree. The more positive SES.MPD values indicate that species are more dispersed in the phylogenetic tree and the more negative SES.MPD values that species are closer in the phylogenetic tree (Appendix 1). Values for high phylogenetic diversity scenarios (PSV = 0.82 and 0.85; SES.MPD = 0.53 and 0.17) were decided on the basis of those naturally observed in the field (PSV ~ 0.8; data from Ref.^12^), while values for low diversity assemblages were experimentally diminished by choosing related species of the family Asteraceae (PSV = 0.24; SES.MPD = − 9.6; p < 0.001) and species of the orders Brassicales and Malvales (PSV = 0.64; SES.MPD = − 2.5; p < 0.05). These indices were calculated running the R packages “ape”^57^ and “picante”^58^ based on the phylogenetic tree for the 28 species involved in the experiment built using “V.Phylomaker” package, using *phylo.maker* function and the “scenario1” option to bind new tips^59^ (Fig. 2). To control for the idiosyncratic effect of species identities, we established two different species combinations for each phylogenetic diversity level. Thus, four taxonomic combinations were constructed comprising two combinations of distantly related species (high phylogenetic diversity scenarios) and two of more closely related species (low phylogenetic diversity scenarios). High phylogenetic diversity scenarios were composed of distantly related species such as members of the Poaceae, Crassulaceae, Apiaceae, Caryophylaceae families (see Fig. 2) that are known to have contrasting life strategies. Specifically, *Pistorinia hispanica* is known to have CAM metabolism, species of the Poaceae family usually develop fasciculate roots, some species in these scenarios are rosette forming plants (i.e. *Torilis leptophylla*, *Campanula erinus*, *Limonium echioides*), while others do not form rosettes (*Ziziphora hispanica*, *Silene conica* or *Lomelosia stellata*), some species maximum plant heights are around 40 mm (*Echinaria capitata*, *Plantago afra*, *Campanula erinus*), while others can grow above 150 mm (*Torilis nodosa* and *T. leptophylla*, for example). In our high phylogenetic diversity scenarios, there are species with contrasting seed mass values (i.e. seeds below 20 µg of *Pistorinia hispanica* or *Campanula erinus* to seed mass values of 1 mg for *Neatostema apulum* or 1.4 mg for *Lomelosia stellata*, for example).

We established water availability treatments with two levels in a fully crossed factorial design: average precipitation vs. drought. The average precipitation treatment simulated the monthly average rainfall recorded between 1981 and 2010 in the study area, and the drought treatment used 33% of the average rainfall for each month**.** We established two phylogenetic diversity levels × two taxonomic combinations of species × two water availability treatments (eight experimental scenarios). Each scenario was replicated in 10 to 16 units, thereby resulting in 110 experimental assemblages.

A common garden experiment was conducted in a greenhouse at Rey Juan Carlos University (https://urjc-cultive.webnode.es/ Móstoles, Madrid, Spain: 40° 20′ 2″ N, 3° 52′ 00″ W, 650 m) from October 2017 when the seeds were sown, until June 2018 when the last individuals were collected. We used round pots with a diameter of 30 cm and height of 10 cm, which were filled with seed-free gypsum soil from a gypsum quarry located close to the collection sites. We aimed to establish 10 plants of each seven coexisting species per pot, so we initially sowed 70 seeds per species in each one. Excess emergent seedlings were removed every two days trying to avoid clusters of seedlings to ensure the planned abundance of each species. By this way, we got to reproduce high densities of ephemeral, highly synchronized annual plants (i.e., around one plant per each 10 cm^2^ in average) in our experimental assemblages, close to those often observed in the field, thus promoting the full activation of the interaction net among participant plants. We watered pots to the soil water-carrying capacity for the first 20 weeks to ensure the establishment of experimental assemblages at the emergence stage mimicking natural field conditions and then commenced the water availability treatments, which were maintained for 19 weeks. Between February and June, we monitored plant survival per species and per pot (summing 7700 plants) every two weeks, and we recorded the numbers of flowering plants once a week. In addition, for each species and pot we registered the final number of plants that reached the fruiting stage.

Generalized linear mixed models (GLMMs) were employed to analyze the proportion of surviving, flowering, and fruiting plants per species and pot (Table 1; Appendix 2) and to evaluate the overall proportion of species and plants that survived per pot (Table 2; Appendix 2). We used the irrigation treatment (2 levels: average and drought) and the initial phylogenetic diversity (2 levels: high and low PD) as fixed factors and we included the interaction term between both. Pot identity (n = 110) and taxonomic composition (4 combinations of species were set up in the experimental design: 2 species combinations with high PD and 2 with low PD) were included in the models as random factors. Sampling moment (Time) was considered as the number of days since the beginning of the experiment and used as a covariate (n = 10 for surviving plants and n = 19 for flowering plants). We did not consider the sampling moment to model the proportion of fruiting plants, because this variable was the percentage of the total cumulative number of fruiting plants per species in each pot. We used the “glmer” function in the “lme4” package with a binomial error distribution and the logit link function All statistical procedures were performed in R (4.0.3 version) (R Core Team, 2020).

**Table 1:** Generalized linear mixed models (GLMMs) for the proportion of surviving, flowering and fruiting plants per species and pot.

Fixed effects	Df	Proportion of surviving plants per species (n = 7700)				Proportion of flowering plants per species (n = 14,626)				Proportion of fruiting plants per species (n = 769)			
		Coef (± SE)	CI 2.5	CI 97.5	Wald Chisq	Coef (± SE)	CI 2.5	CI 97.5	Wald Chisq	Coef (± SE)	CI 2.5	CI 97.5	Wald Chisq
Intercept	1	0.84 (± 0.41)	− 0.2	1.9	4.26*	− 1.8 (± 0.2)	− 2.4	− 1.3	77.0***	1.4 (0.2)	0.9	1.9	47.8***
Time	1	− 3.55 (± 0.03)	− 3.6	− 3.5	19,107.6***	− 0.52 (± 0.01)	− 0.54	− 0.51	3656.0***	–	–	–	–
PD	1	− 0.68 (± 0.57)	− 2.1	0.8	1.42^ns^	− 0.32 (± 0.3)	− 1.1	0.4	1.1^ns^	0.08 (± 0.29)	− 0.6	0.8	0.07^ns^
W	1	− 0.97 (± 0.07)	− 1.1	− 0.8	206.5***	− 0.37 (± 0.05)	− 0.5	− 0.26	44.9***	− 1.4 (± 0.14)	− 1.7	− 1.1	109.0***
PD × W	1	− 0.53 (± 0.1)	− 0.7	− 0.3	27.9***	− 0.44 (± 0.08)	− 0.6	− 0.3	28.5***	− 0.6 (± 0.2)	− 1.0	− 0.2	8.8**

Dependent variables were modelled using binomial error distributions and logit link functions. Pot identity (n = 110) and taxonomic composition (n = 4) were included in the model as random factors. Sampling moment (time) was considered as the number of days since the beginning of the experiment and used as a covariate (n = 10 for surviving plants and n = 19 for flowering plants). We did not consider the sampling moment to model the proportion of fruiting plants, because this variable was just the percentage of the total cumulative number of fruiting plants per species in each pot. Phylogenetic diversity (PD) and water availability (W) were used as fixed factors. Type III Wald Chi-square tests were performed to estimate significance. Number of observations are shown for each dependent variable.

*Coef*. Coefficient, *Df* degrees of freedom, *SE* standard error, *CI* confidence interval, *ns* not significant.

*p < 0.05; **p < 0.01; ***p < 0.001.

**Table 2:** Generalized linear mixed models (GLMMs) for the proportion of surviving species and proportion of total plants per pot.

Fixed effects	Df	Proportion of surviving species per pot (n = 1100)				Proportion of surviving plants per pot (n = 1100)			
		Coef (± SE)	CI 2.5	CI 97.5	Wald Chisq	Coef (± SE)	CI 2.5	CI 97.5	Wald Chisq
Intercept	1	1.6 (± 0.2)	1.0	2.1	50.4***	0.76 (± 0.4)	− 0.3	1.8	3.4^ns^
Time	1	− 4.1 (± 0.1)	− 4.3	− 3.9	1717.7***	− 3.4 (± 0.02)	− 3.4	− 3.3	19,778.4***
PD	1	− 0.6 (± 0.3)	− 1.4	0.2	3.7^p=0.055^	− 0.7 (± 0.6)	− 2.1	0.8	1.3^ns^
W	1	− 0.9 (± 0.1)	− 1.1	− 0.7	58.3***	− 0.9 (± 0.07)	− 1.1	− 0.8	173.8***
PD × W	1	− 0.6 (± 0.2)	− 0.9	0.3	12.4***	− 0.5 (± 0.1)	− 0.7	− 0.3	25.2***

Dependent variables were modelled using binomial error distributions and logit link functions. Pot identity (n = 110) and taxonomic composition (n = 4) were included in the model as random factors. Sampling moment (time) was considered as the number of days since the beginning of the experiment and used as a covariate (n = 10) to statistically control for the effect of time. Phylogenetic diversity (PD) and water availability (W) were used as fixed factors. Type III Wald Chi-square tests were performed to estimate significance. Number of observations are shown for each dependent variable.

*Df* degrees of freedom, *Coef.* Coefficient, *SE* standard error, *CI* confidence interval, *ns* not significant.

*p < 0.05; **p < 0.01; ***p < 0.001.

### Ethical statement

Authors assure that legislation on seed collection has been accomplished. Permission obtained from responsible authority to collect seeds.

## Results

**Figure 3:**
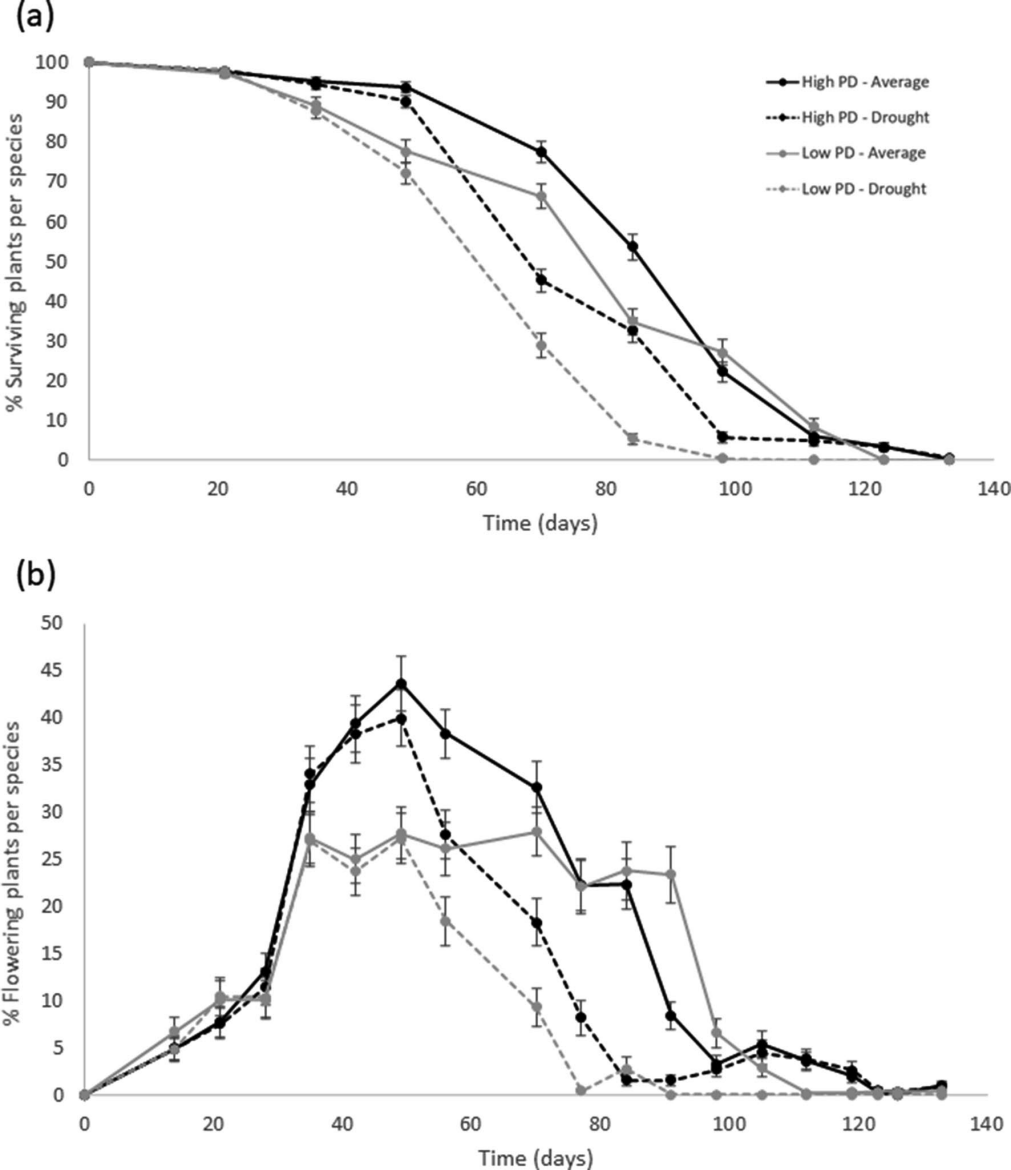
(**a**) Percent of surviving plants and (**b**) percent of flowering plants per species and per pot on each sampling date (see Table 1). Black lines represent high phylogenetic diversity (PD) scenarios and grey lines denote low phylogenetic diversity scenarios. Solid lines represent the average precipitation treatments based on natural precipitation for 30 years, and spotted lines denote drought treatments (33% of the average precipitation). Vertical bars represent the standard error.

**Figure 4:**
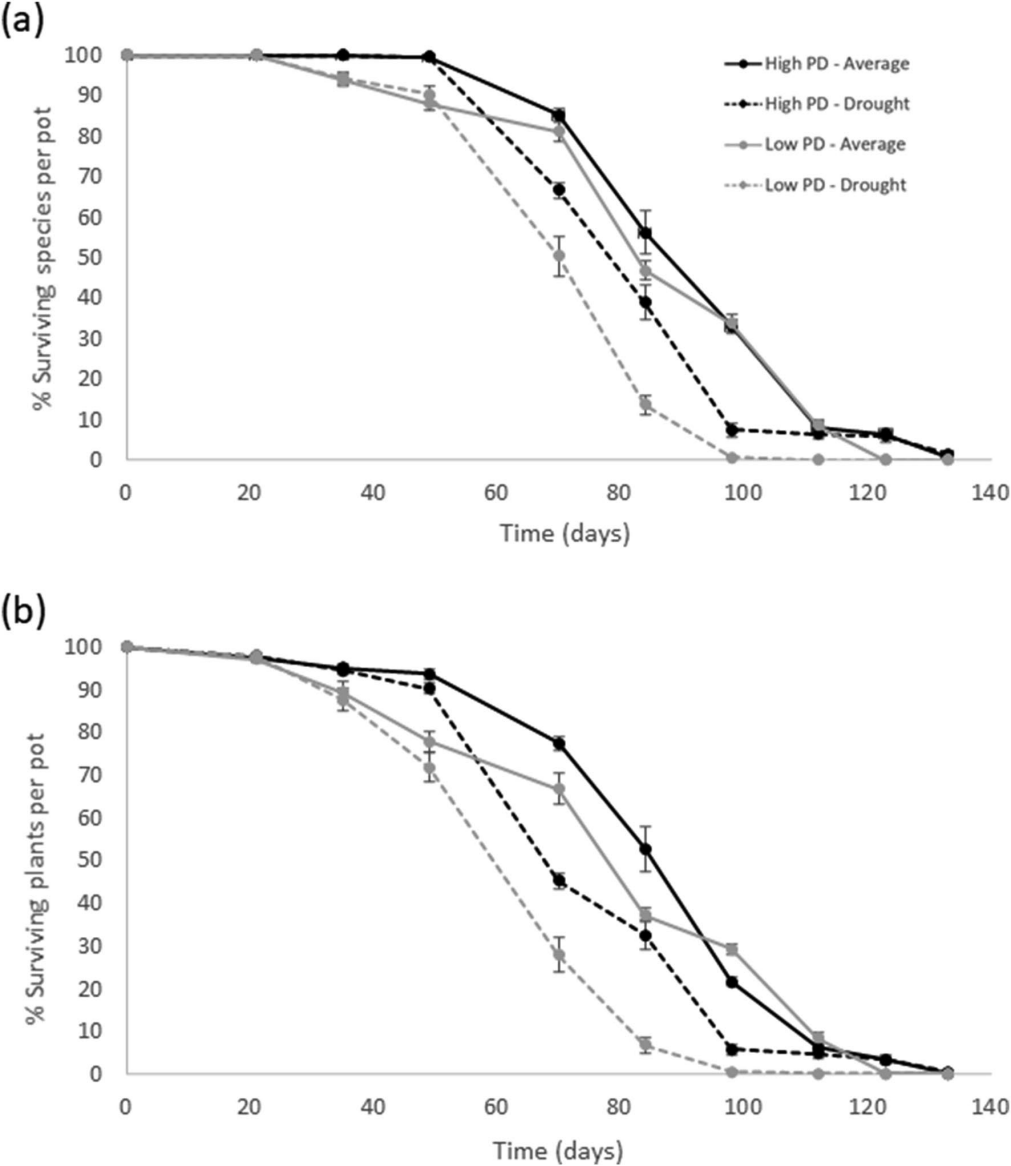
(**a**) Percent of surviving species per pot and (**b**) percent of surviving plants per pot on each sampling date (see Table 2). Black lines represent high phylogenetic diversity (PD) scenarios and grey lines denote low phylogenetic diversity scenarios. Solid lines represent the average precipitation treatments based on natural precipitation for 30 years, and spotted lines denote drought treatments (33% of the average precipitation). Vertical bars represent the standard error.

**Figure 5:**
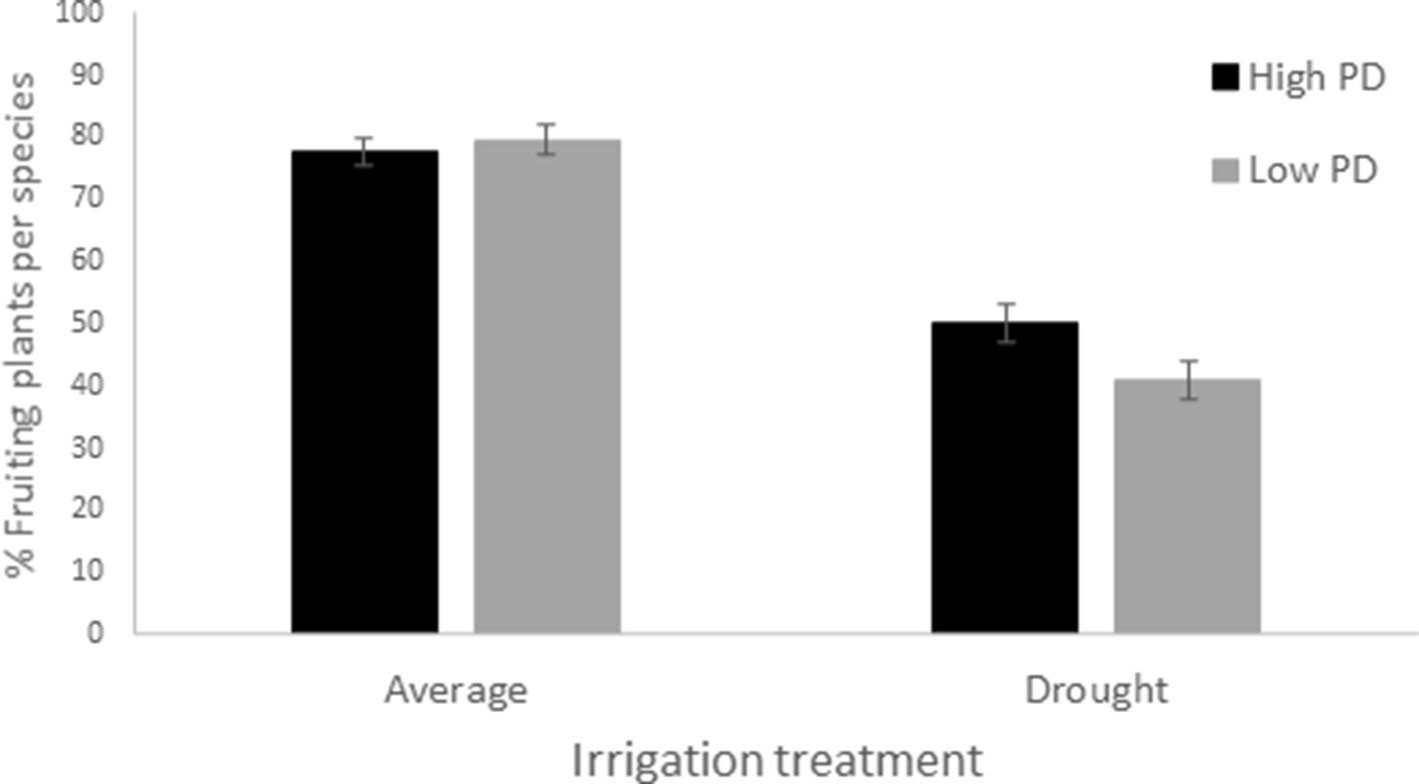
Percent of fruiting plants per species and pot (see Table 1). Black bars represent high phylogenetic diversity scenarios and grey bars low phylogenetic diversity scenarios. Average precipitation treatments are based on natural precipitation for 30 years in the field and drought treatments represent 33% of the average precipitation. Vertical bars represent the standard error.

The annual plant species that formed the experimental assemblages completed their life cycle within 5 months (Fig. 3a). Plant mortality concentrated between the 2nd and the 3rd month of the experiment since plants died shortly after fruit maturation. Flowering started in the first weeks of the experiment and lasted for nearly four months (Fig. 3b). Our results showed that under low water conditions (33% of the average precipitation treatment), phylogenetically more diverse assemblages favored species coexistence. In particular, in low water conditions, we found that the experimental assemblages formed of distantly related species resulted in more surviving plants per species (Table 1, Fig. 3a) and more species coexisting in each pot along time (Table 2; Fig. 4a) In addition, under dry experimental conditions more plants flowered (Fig. 3b) and fructified (Fig. 5) per species in distantly related assemblages compared with those that were closely related. Furthermore, plant survival (regardless of species identity) was higher in high phylogenetic diversity assemblages under drought conditions (Fig. 4b). Consequently, the experimental assemblages with high phylogenetic diversity were less sensitive to drought than the low phylogenetic diversity assemblages in terms of the plant survival, number of coexisting species, and numbers of flowering and fruiting plants in each experimental unit.

## Discussion

As hypothesized, phylogenetic relatedness among coexisting plants drives community level processes such as survival and reproduction. In particular, we demonstrated the higher resistance of phylogenetically diverse assemblages to drought in terms of plant survival and number of coexisting species over time, and even more, plants not only were able to survive more successfully to drought in phylogenetically diverse assemblages, but also more individuals completed the reproductive stage by setting flowers and fruits. Overall, these results support the idea that phylogenetic relatedness predicts niche differences among species (Hypothesis 1a in Fig. 1), and that drought resistance might be convergent along phylogeny (Hypothesis 2b in Fig. 1).

Our results are consistent with the species niche complementarity concept^4,60^, which predicts that species with differences in terms of their resource use are more likely to coexist due to the reduced competitiveness among them^22,60–62^. Distantly related species are more likely to be phenologically and functionally complementary, and thus to suffer less from the effects of competition compared to living among conspecifics or close relatives in the neighborhood^63–65^. Closely related species are likely to have ecologically similar requirements^66,67^, so they would share fundamental niches and be more prone to compete strongly for resources. Several mechanisms could promote niche complementarity, such as phenological differences among species^68,69^, different resource use traits^70–72^, or different root foraging activities^73^. Maynard et al.^74^ found that phenological differences among the species in a community could affect the competitive dynamics to promote coexistence, thereby possibly leading to an increase in species richness at fine spatial scales, which could promote stabilizing dynamics. In our study, the distantly related species probably differed in terms of their phenology, resource uptake, and physiological efficiency, which could have reduced the intensity of the competitive interactions among them^14^, particularly when water availability is limited in low irrigation treatments This situation could have promoted individual plant survival and species richness, as well as higher plant fitness in high phylogenetic diversity assemblages in drought conditions (see also Ref.^75^).

Furthermore, our results support the idea that drought resistance is a convergent strategy along phylogeny of annual plant species in our study system (Hypothesis 2b in Fig. 1). Our results also agree with García-Camacho et al.^76^ who did not detect phylogenetic conservatism in terms of the rainfall preferences of 111 annual plant species from an aridity gradient in Israel. Thus, the annual plant species in dryland areas have evolved over a long period under strong pressure due to drought events and highly unpredictable rainfall events, which might have resulted in the convergent adaptation of distantly related phylogenetic clades to cope with limiting water conditions. Clearly, a powerful abiotic filter such as severe droughts could have shaped the regional species pool over an evolutionary time scale. Consequently, regardless of phylogenetic relatedness, all the species in the community would be able to cope with water limitation, including when it occurs over an ecological time scale^77^. Nevertheless, our results could also concur with the stress gradient hypothesis, which states that facilitation is more relevant than competition for the assembly of species under stressful abiotic conditions^13^. In that case, the clades adapted to withstand drought could have improved the micro-environmental conditions in their close neighborhoods, thus favoring survival and fertility of distantly related less tolerant clades^35^. However, to the best of our knowledge, this has not been tested with annuals at the small spatial and time scales monitored in our experimental setup, where all the species are very small (less than 15 maximum height) and had very synchronous life cycles in a relatively short time lapse (between six and nine months depending on the species) (but see Ref.^78^, for positive plant-plant interactions with a large annual nurse plant in a field study).

Assembly processes that occur at the community level may affect the evolution of species over the long term (see also Refs.^21,79,80^). Coexistence among closely related species can trigger character displacement to reduce competition intensity^81^ or character convergence to reduce competition asymmetry^52^. Community processes seem to exert crucial effects on evolution^79^, and provide a plausible explanation for the intriguing question regarding how so many annual plant species can coexist in the harsh conditions of semiarid gypsum systems. Based on this, we suggest that the species assembly occurring at the finest spatial scales where species interact, could a be major driver of the high taxonomic diversity observed at larger spatial scales in our study system (more than 120 annual species^43^). In this line, our findings support the “united we stand” framework^82^, which states that rare species might remain in assemblages by establishing diffuse positive interactions. Such “diffuse positive interactions” could be related to high phylogenetic diversity among neighboring plants at fine spatial scales (see Ref.^83^).

In conclusion, our results agree with the existence of niche complementarity and the evolutionary convergence of water economy strategies as major mechanisms for organizing annual plant assemblages in semiarid Mediterranean gypsum habitats. Species that grow in assemblages of distantly related species are more likely to survive and fructify in drought conditions than those that grow in closely related ones. Intense droughts occur often in semiarid Mediterranean ecosystems^12,47^ and their intensity is expected to increase in the future^84^. Furthermore, the United Nations recently declared the next decade as “The Decade on Ecosystem Restoration” (United Nations Environment Programme, 2019), and they remarked the importance of understanding the drivers of community assembly. In this context, our study is timely since restoration strategies are moving beyond traditional restoration actions and adopting new tools that better describe the characteristics of species assemblies. In this way, we demonstrate that phylogenetic diversity is an excellent measure that can be used to understand species assembly processes. Furthermore, our study highlights that experimental approaches can provide new answers to old questions in community ecology by connecting assembly processes and patterns in a more robust causal framework. We show that species rich annual plant communities are excellent model systems for such investigations, due to the feasibility of manipulating species assemblages and the short time lapses needed to account for a complete generation.

## Supplementary Information

Supplementary Information.
