# Supplementary material for: Diverse phylogenetic neighborhoods enhance community resistance to drought in experimental assemblages
Source: Sci Rep. 2021 Nov 18;11:22499. doi: 10.1038/s41598-021-01991-z (PMC8602379; doi:10.1038/s41598-021-01991-z)
Supplement: Supplementary file 1 — Supplementary Information. [file 41598_2021_1991_MOESM1_ESM.docx]

**Appendix 1.** Species compositions in the experimental assemblages. Two species combinations were prepared for high phylogenetic diversity scenarios and two for low phylogenetic diversity scenarios. The phylogenetic tree was built using the “*V.PhyloMaker*” package and the phylogenetic indices with “*picante*” package in R. PSV: Phylogenetic species variability. MPD.SES: Standardized Mean Pairwise Distance.

| **High phylogenetic diversity scenario**  **(PSV = 0.82; MPD.SES = 0.17)** | **High phylogenetic diversity scenario**  **(PSV = 0.85; MPD.SES = 0.53)** | **Low phylogenetic diversity scenario**  **(PSV = 0.24; MPD.SES = -9.6)** | **Low phylogenetic diversity scenario**  **(PSV = 0.64; MPD.SES = -2.53)** |
| --- | --- | --- | --- |
| *Echinaria capitata* (L.) Desf.  (Poaceae) | *Brachypodium distachyon*(L.) P. Beauv.  (Poaceae) | *Asteriscus aquaticus*(L.) Less.  (Asteraceae) | *Alyssum simplex*Rudolphi  (Brassicaceae) |
| *Plantago afra* L.  (Plantaginaceae) | *Torilis nodosa* (L.) Gaertn.  (Apiaceae) | *Senecio gallicus*Vill.  (Asteraceae) | *Clypeola jonthlaspi* L.  (Brassicaceae) |
| *Torilis leptophylla* (L.) Rchb. fil.  (Apiaceae) | *Campanula erinus*L.  (Campanulaceeae) | *Centaurea melitensis*L.  (Asteraceae) | *Linum trigynum*L  (Linaceae) |
| *Pistorinia hispanica* (L.) DC.  (Crassulaceae) | *Neatostema apulum*(L.) I.M. Johnst.  (Boraginaceae) | *Leontodon taraxacoides* (Vill.) Mérat  (Asteraceae) | *Malva aegyptia*L.  (Malvaceae) |
| *Bartsia trixago* L.  (Orobanchaceae) | *Lomelosia stellata* (L.) Raf.  (Dipsacaceae) | *Crupina vulgaris*Pers. ex Cass.  (Asteraceae) | *Reseda stricta*Pers.  (Resedaceae) |
| *Parentucellia latifolia*(L.) Caruel  (Orobanchaceae) | *Ziziphora hispanica*L.  (Lamiaceae) | *Filago pyramidata*L.  (Asteraceae) | *Helianthemum ledifolium*(L.) Mill.  (Cistaceae) |
| *Silene conica*L.  (Caryophyllaceae) | *Limonium echioides* (L.) Mill.  (Plumbaginaceae) | *Bombycilaena discolor*(Pers.) M. Laínz  (Asteraceae) | *Helianthemum salicifolium*(L.) Mill.  (Cistaceae) |

**Appendix 2**. **R scripts of the five statistical models performed in this study**. Abbreviations: *surv/initial*: proportion of surviving plants of each species in each pot in each sampling moment. *flowpl/initial*: proportion of flowering plants of each species in each pot in each sampling moment. *fruitpl/initial*: proportion of the final number of fruiting plants of each species in each pot. *surv_plants/initial_plants*: proportion of surviving plants per pot in each sampling moment. *surv_sp/initial_sp*: proportion of surviving species per pot in each sampling moment. *PD*: Phylogenetic diversity treatment. *Time*: time since the beginning of the experiment. *Taxoncomp*: taxonomic composition (i.e. two different species composition with high PD and two different species composition with low PD)

2 different species compositions for high PD and 2 for low PD).

At the species level:

*Model1<- glmer (surv/initial ~ PD*irrigation + Time +(1|taxoncomp) + (1|pot), weights= initial, family=binomial, data= DATOS1)*

*Model2<- glmer (flowpl/initial ~ PD*irrigation + Time +(1|taxoncomp) + (1|pot), weights= initial, family=binomial, data= DATOS2)*

*Model3<- glmer (fruitpl/initial ~ PD*irrigation + Time +(1|taxoncomp) + (1|pot), weights= initial, family=binomial, data= DATOS3)*

At the pot level:

*Model4 <- glmer (surv_plants/initial_plants ~ PD*irrigation + Time +(1|taxoncomp) + (1|pot), weights= initial, family=binomial, data= DATOS4)*

*Model5 <- glmer(surv_sp/initial_sp ~ PD*irrigation + Time +(1|taxoncomp) + (1|pot), weights= initial, family=binomial, data= DATOS5)*
